# Clinical findings of candidate stallions presented for licensing at all German Warmblood horse‐breeding associations in 2018–2020

**DOI:** 10.1111/evj.14474

**Published:** 2025-01-22

**Authors:** Muriel Sarah Folgmann, Kathrin Friederike Stock, Karsten Feige, Uta Delling

**Affiliations:** ^1^ Clinic for Horses University of Veterinary Medicine Hannover Hannover Germany; ^2^ IT‐Solutions for Animal Production Verden Germany

**Keywords:** clinical examination, database, horse, horse health monitoring, prevalence, stallion licensing

## Abstract

**Background:**

There is very little information available about the health status of young stallions from the German Warmblood population that will, once licensed, shape the future of equestrian sport and horse breeding.

**Objectives:**

To evaluate the prevalence of clinical findings at licensing examinations of candidate stallions and the influences of season of birth, age at licensing, year of licensing, and the evaluator on the distribution of recorded findings.

**Study design:**

Retrospective observational study.

**Methods:**

Clinical records of 1655 candidate stallions presented for licensing in 2018–2020 were reviewed. Data were provided by all German Warmblood horse‐breeding associations and their official veterinarians. Storage and processing of the records was performed using the German equine health database. Generalised linear models were used to determine the influences of fixed effects (season of birth, age at licensing, year of licensing, evaluator) on main clinical findings. The significance level was set at *p* < 0.05.

**Results:**

No remarks were documented on the clinical examination protocol in 777 of the 1655 horses (47.0%). Furthermore, 51.9% of those stallions with remarks had only one finding documented. The main abnormalities recorded were skin lesions, enlargements on the limbs, and testicular findings. The distributions of several clinical findings differed significantly between the evaluators.

**Main limitations:**

Homogenous study population and retrospective data.

**Conclusions:**

The clinical part of the licensing examination of German Warmblood candidate stallions presented in 2018–2020 rarely revealed abnormalities. The majority of the clinical findings which were recorded are considered to be of minor clinical relevance, implying an overall favourable clinical health status of the presented stallions.

## 1 INTRODUCTION

German Warmblood breeding is, except for the Trakehner, characterised by open studbooks, enabling free exchange of genetic material across the distinct breeds.^1^ Accordingly, the German horse‐breeding associations have, co‐ordinated by their umbrella organisation, the German Equestrian Federation (FN), and in collaboration with their official veterinarians, promoted the idea of a joint equine health database in Germany to maintain and advance their common values.^2^, ^3^ The database has been available since 2014 and allows standardised, online data recording. The database aims to cover all types of equine examination data but functionalities for documentation are currently confined to examinations of candidate stallions performed prior to licensing.^4^

Licensing is obligatory for stallions intending to start a breeding career. The responsibility for licensing rests with the individual breeding associations, but there are some general requirements that must be fulfilled by all German Warmblood stallions: (1) The age of the stallion when presented for licensing must be a minimum of 2 years at evaluation, (2) the stallion must participate in a preselection if offered by the respective breeding association, and (3) the stallion must not have any health condition defined as disqualifying for breeding use.^5^ The latter is verified by the veterinary medical licensing examination performed prior to the stallion's licensing. This examination consists of a clinical examination, which is the subject of this article, and a radiological examination of the limbs. The veterinary medical licensing examinations are performed by official veterinarians according to the German Breeding Association Regulations (‘*Zuchtverbandsordnung*’).^6^ The outcome of the licensing examination is documented and submitted to the respective breeding associations. The licensing process, performed by a distinct commission of the breeding association, includes evaluations of all stallions' conformation, movement, and, in some cases, jumping ability. A licence only grants limited breeding permission. In order to switch from temporary to permanent approval for breeding use, stallions must fulfil further performance‐related criteria referring to the outcome of standardised performance tests or successful participation in equestrian sport.^7^ Accordingly, the health status of the individual stallions plays an important role in the process of licensing, but stallion selection is currently not based on distinct health traits. Studies have shown that the variation in the genetic disposition to health conditions in Warmblood horses is reasonable and appropriate for use in breeding, but it has been difficult to access and assess health data.^8^ Current electronic studbook‐keeping covers health‐related information only sparsely, not because no data are collected, but because respective data are mostly neither standardised nor accessible.^9^, ^10^ Consequently, there are no comprehensive studies available about the outcome of clinical examinations of young Warmblood horses in Germany. Previous research focused on radiographic findings^11^, ^12^, ^13^ or certain clinical conditions assessed in heterogeneous groups of horses rather than distinct breeding populations.^14^, ^15^

This study is part of a large project in which the German equine health database has been used for data collection and analysis for the first time. The aim of the project was to specifically determine the prevalences of clinical and radiological findings of all German Warmblood candidate stallions presented for licensing in 2018–2020, and evaluate the influences of age at licensing, year of licensing, season of birth, and the evaluator on their distributions. The current study focuses on the clinical findings; data regarding radiological findings will be presented in a separate publication. We hypothesised that the majority of stallions presented for licensing would not have any clinical abnormalities recorded and that the role of environmental effects on the distribution of clinical findings would be mostly minor.

## 2 MATERIALS AND METHODS

### 2.1 Study design

Data available for this study were the results of the clinical part of the examinations of candidate stallions prior to the licensing of German Warmblood horses in 2018–2020.

All data which were provided by the breeding associations responsible for German Warmblood horses were entered by the first author into the German equine health database to make them centrally available for analyses. The data were supplied in analogue form, that is, handwritten or typed on a computer, as printed paper or scanned digital files. Data recording into that database was performed thoroughly and if needed, in consultation with the responsible veterinarians, by a single researcher (MSF) in 2021–2022 using the web‐based interface ‘serv.it VET’, which offers standardised entry options and additional free text fields if needed.^4^

**FIGURE 1:**
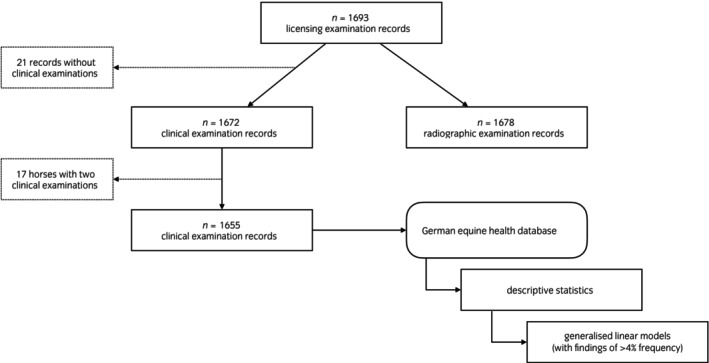
Flow chart documenting the flow of information through the study design.

The clinical examinations were performed by the official veterinarians or external equine practitioners according to the protocol published as part of the German Breeding Association Regulation (Figure S1).^6^ All recorded results in section 8 to 24 were used in this study. Additionally, stallions were identified in the system by their universal equine life number (UELN, section 3) and information of ‘date of birth’ (section 2), ‘previous diseases/surgeries’ (section 5), ‘medications within the last 6 weeks’ (section 5) and ‘vaccination according to horse passport’ (section 6) were included. Each examination protocol of an external equine practitioner was approved by the respective official veterinarian and these official veterinarians are uniformly referred to as evaluators in this study. When multiple examinations were available for one horse, for example, when stallions were presented at more than one licensing examination and health data were updated, only the results of the most recent examination were included in the analyses (Figure 1). Omissions in single parts of the clinical examination were treated as missing information and resulted in different denominators in the results. Age at clinical examination, referred to as licensing age, was calculated using the examination and birth dates.

### 2.2 Data analysis

Clinical examination data were exported for statistical analyses. The software package SAS® (Statistical Analysis System, version 9; SAS Institute) was used for data processing, which included plausibility checking and possible editing of the health data, as well as for generating descriptive statistics and performing generalised linear models.

Frequencies of clinical findings were determined based on all documented findings and grouped on different hierarchical levels up to organ systems. Basic distribution figures were obtained using the SAS procedures FREQ and MEANS. Influences on the distributions of clinical findings with a frequency >4% were subsequently investigated by generalised linear models using the SAS procedure GENMOD accounting for the non‐linear nature of the analysed traits. Following the threshold theory, the discontinuous trait expressions depend on whether or not an underlying continuous liability has passed a certain threshold. Accordingly, binomial distribution and probit link functions were used for binary traits, and categorical distribution and cumprobit link functions were used for traits with more than two distinct levels of expression. Environmental factors were tested for their possible impact on the distributions of the selected subset of clinical findings. Model development considered the results of the standard model fit statistics and aimed at the most parsimonious model suitable to be used across all traits. The final model included a season of birth, age at licensing (in classes), year of licensing and evaluator as fixed effects. Evaluators with relatively few observations (<7% of all data) were grouped together to allow a reliable assessment of this fixed effect.

$$y_{\text{ijklm}}=\mu +{\mathrm{Age}}_{i}+{\text{Year}}_{j}+{\text{BSeason}}_{k}+{\text{Evaluator}}_{l}+e_{\text{ijklm}},$$

where *y*_*ijklm*_ = clinical finding, *μ* = model constant (overall mean), *Age*_*i*_ = fixed effect of the *i*‐th age at licensing (*i* = 1–3; <30, 30–36, >36 months of age), *Year*_*j*_ = fixed effect of the *j*‐th year of licensing (*j* = 1–3; 2018, 2019, 2020), *BSeason*_*k*_ = fixed effect of the *k*‐th season of birth (*k* = 1–5; November to February, March, April, June, July to October), *Evaluator*_*l*_ = fixed effects of the *l*‐th evaluator (*l* = 1–6; five evaluators individually, three evaluators grouped together), and *e*_*ijklm*_ = random residual.

Least square mean estimates (LSM) were transformed from the underlying normal liability scale to the observed scale and determined with standard errors and 95% confidence intervals. Accordingly, the LSM are expressed on the same scale as the traits were recorded, such that they reflect the expected average frequency of occurrence for binary traits (e.g., 0.11 on 0/1‐scale corresponding to 11%) and the expected average trait value for categorical traits with more than two levels. The level of significance was set at *p* < 0.05.

## 3 RESULTS

### 3.1 General results

**FIGURE 2:**
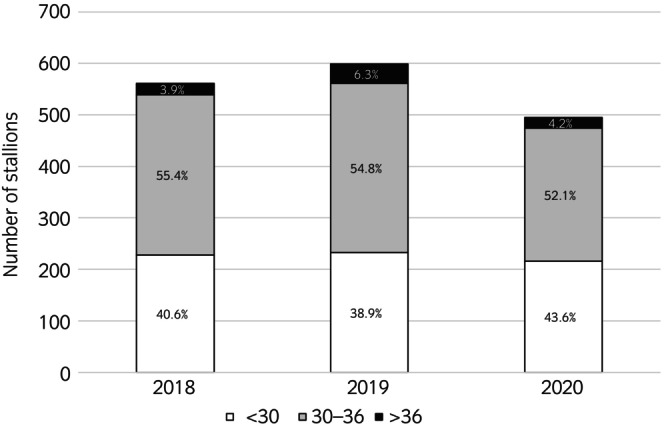
Distribution of age groups at licensing (in months) per year of licensing from 1655 examinations of candidate stallions prior to licensing for German Warmblood breeding in 2018–2020.

A total of 1655 clinical examinations approved by eight evaluators were available for analysis (Table 1). At licensing, 40.9% (677/1655) of the stallions were less than 30 months old at the time of the licensing examination (Figure 2). Previous surgeries were mentioned in 120/1655 (6.8%) records, with the majority (94.2%, 113/120) specified as orthopaedic surgeries and involving the fetlock joint (62.8%, 71/113), tarsal joint (36.3%, 41/113) and coffin joint (7.1%, 8/113). The remaining 6/120 (5.0%) surgical sites were the umbilical region, teeth, injuries, or hoof abscesses. Information on medications within the last 6 weeks was given in 1479 examination and 87.2% (1290/1479) of the horses had no medication during that time period. The most commonly documented medication was sedation (57.7%, 109/189), with a selection of drugs (detomidine, romifidine, xylazine, acepromazine). Some type of vaccination was recorded for 88.6% (1467/1655) of the stallions: equine influenza virus 99.1% (1454/1467), tetanus 97.8% (1435/1467) and equine herpesvirus 50.2% (737/1467). The most frequently recorded combinations of vaccinations were equine influenza, tetanus, and equine herpes (49.4%, 725/1467), and equine influenza and tetanus (47.6%, 698/1467).

**TABLE 1:** Distribution of the German Warmblood candidate stallions (*n* = 1655) by environmental factors with absolute (*n*) and relative frequencies (%).

Environmental factor		*n*	%
Year of licensing	2018	561	33.9
	2019	599	36.2
	2020	495	29.9
Year of birth	<2015	6	0.4
	2015	109	6.6
	2016	578	34.9
	2017	565	34.1
	2018	397	24.0
Season of birth	November–February	121	7.3
	March	315	19.0
	April	514	31.1
	May	528	31.9
	June–October	177	10.7
Age at licensing (in months)	<30	677	40.9
	30–36	897	54.2
	>36	81	4.9
Evaluator	1	313	19.0
	2	230	13.9
	3	221	13.4
	4	293	17.7
	5	317	19.2
	6	86	5.2
	7	114	6.9
	8	80	4.8
Breed	Hanoverian	433	26.2
	Holsteiner	272	16.4
	Oldenburg	215	13
	Westphalian	212	12.8
	German Sport Horse	139	8.5
	Oldenburg International	125	7.6
	Trakehner	105	6.3
	Royal Dutch Sport Horse	53	3.2
	Zangersheide Studbook	30	1.8
	Mecklenburger	24	1.5
	Others (<20)	47	2.8

### 3.2 Clinical findings

The clinical examination was recorded as unremarkable in 777 horses (47.0%). In the remaining 53.1% (878/1655) of horses, up to 10 findings per horse had been documented. However, more than half of them (51.9%, 456/878) had one finding only. Among the horses with multiple findings, these were not necessarily distributed over different body structures or organ systems but could also refer to distinct types of finding within one body structure or organ system.

Musculoskeletal findings were recorded most frequently. Enlargements (exostoses or swellings or fillings) on the limbs were seen in 234/1653 horses (14.2%), with a slightly higher proportion on the forelimbs (9.1%, 150/1653) than the hindlimbs (6.4%, 105/1653). The metacarpal/metatarsal bone and adjacent splint bones were mentioned most often (9.8%, 162/1653; front: 7.4%, 123/1653; hind: 2.9%, 48/1653), followed by the pastern region (2.4%, 40/1653) and the tarsus (1.9%, 32/1653). Exostoses were mentioned specifically on 158/1653 metacarpal/metatarsal bones (9.6%) and were more common in the front limbs (7.4%, 123/1653). Joint filling was rarely recorded, with the tarsal joints (1.3%, 21/1653) and the fetlock joints (0.5%, 9/1653) as the main locations. Capped hocks were mentioned in 14/1653 (0.8%) examinations. Abnormal limb conformation was noted in 93/1587 (5.9%) examinations, of which 54 stallions were described as toed‐out (front: 83.3%, 45/54) and 35 stallions as toed‐in (front: 94.3%, 33/35). Muscular indentations were documented in 2.4% (40/1650) of the horses, mainly in the neck (1.6%, 26/1650). Skin lesions (scars or injuries) were documented in 12.5% (206/1654) examinations and mainly in the lower limbs (90.8%, 187/206). The majority, 93.3% (1543/1654) of the candidate stallions were shod in the front feet.

The standardised protocol requires comprehensive documentation of external genitalia in all horses. Testicular size was most commonly described as similar to a goose egg (59.4%, 983/1655) or duck egg (36.7%, 608/1655). Only 1.6% (26/1655) of the stallions were noted to have testicles of the size similar to a chicken egg. Testicular asymmetry was noted in 47 (2.9%), testicular torsion of 180° in 47 (2.8%), and soft testicular consistency in 20 (1.2%) of the 1655 examinations.

Respiratory noise was noted in 85/1653 (5.1%) stallions. Laryngoscopy, which is obligatory when abnormal respiratory is found, was performed in 96 (5.8%) examinations. Pharyngeal lymphoid hyperplasia was recorded in 79/96 (82.3%) and asynchronous arytenoid cartilage movement in 18/96 (18.8%) examinations. Minor heart murmurs were found in 15/1653 (0.9%) horses. Dental examination of the incisors revealed a minor overjet in 28/1654 (1.7%) cases. A lens opacity was recorded in 11/1654 (0.7%) examinations.

### 3.3 Effects of fixed factors on the main clinical findings

No significant influences of the season of birth, age at licensing, or year of licensing were found for the majority of the clinical findings analysed with generalised linear models (Table S1). However, the season of birth was significantly associated with enlargements on the front limbs and for injuries or scars (*p* < 0.05). Compared with other seasons of birth, injuries or scars were most likely being recorded in horses born in March or April (Table S2). Also, age at licensing was significantly associated with abnormal limb conformation, respiratory noise, and testicular size (*p* < 0.05). Horses between 30 and 36 months of age were more likely to have findings relating to their limb conformation than older ones (*p* = 0.01). Respiratory noise was significantly more likely to be recorded in young horses when comparing the age classes of <30 months versus >36 months (*p* < 0.001). Testicles smaller than the goose or duck egg size were more likely to be documented in younger than older candidate stallions (<30 vs. 30–36 months: *p* = 0.04, <30 vs. >36 months: *p* = 0.007).

The distributions of enlargements on the metacarpal/metatarsal bone, abnormal limb conformation, respiratory noise, and testicular size differed significantly between evaluators (*p* < 0.001). Patterns of least square mean estimates for evaluators varied between these clinical findings. In most cases, the probability of a recorded finding was significantly increased in more than a single evaluator compared with the others (Table S2).

## 4 DISCUSSION

This study provides insights into the occurrence and frequency of clinical findings in young stallions presented for licensing for German Warmblood breeding in recent years. Harmonised licensing examinations of candidate stallions and the broad support of breeding associations allowed the current analyses across associations.

The population consisted mainly of stallions examined around 30 months of age: Licensing age ranged between 28 and 32 months for 90% of the animals. Guidelines of the German Federal Ministry of Food and Agriculture (Bundesministerium für Ernährung und Landwirtschaft) issued in 2020 request sufficient maturity for horses undergoing targeted training and indicate a minimum age of 30 months.^16^ Preparation for the preselection of candidate stallions starts earlier than that, however, since no riding is involved, this early phase has been categorised as ‘familiarisation’ rather than training. Nevertheless, representatives of the German Federal Ministry of Food and Agriculture, animal welfare officers of the federal states, breeding associations, and the FN are currently evaluating general time shifts of the licensing to support different management practices.^17^

Our hypothesis that the majority of candidate stallions would have no recorded clinical findings was not upheld because 53.1% of our study population had remarks. However, the majority of the respective stallions had only a single or very few clinical findings (51.9% had a single finding).

Musculoskeletal findings were the most frequent in the clinical examination records. This is in line with the fact that 61% of insurance claims in German Warmbloods refer to movement‐related diseases,^18^ and that musculoskeletal disorders are responsible for 50%–70% of the culling of riding horses in Sweden and the Netherlands.^19^, ^20^, ^21^ Enlargement of the distal limb was the most frequent finding in the current study, with a more specific description as ‘splint’ given in 158 records (9.6%). This does not exclude the possibility of a larger number of exostoses that were less specifically recorded by the type of finding, limb and location. Splints are mostly seen in young horses undergoing heavy training.^22^ The condition tends to be associated with direct trauma, but conformational abnormalities, such as offset carpi, improper hoof care, and mineral imbalances, may exacerbate it.^23^ In an analysis of pre‐purchase examinations of National Hunt racehorses, 17.1% showed metacarpal/metatarsal exostoses, also with a more frequent affection of the front legs.^24^

Joint filling was specified for 1.3% of hock joints and 0.5% of fetlock joints in these licensing examinations. These results are lower than found in a previous study of pre‐purchase examinations of Warmblood horses, which described minor joint fillings in 15.5% and swellings of the limb in 3.5% of the horses (*n* = 660, mean age = 6.4 years).^13^ The differences may be explained by the different age, sex distribution, and level of training of the current population, and may also reflect different recording procedures.

Limb conformation was noted as abnormal, primarily in the forelimbs, in 5.9% of the records analysed which is in line with previous studies.^8^ The assessment of limb conformation is performed by both the licensing commission and the official veterinarians. It should be noted that the breeding associations expect veterinary comments on limb conformation mainly in cases of obvious clinical relevance, but with some variation between associations. This varying documentation threshold may explain the significant effect of the evaluator on the documented outcome of the assessment of limb conformation.

The testicular examination plays a more important role in licensing examinations than in general clinical or pre‐purchase examinations of Warmblood horses, crucial because of the intended breeding use of the stallions. Therefore, the examination of the male genital tract is one of the sections in the examination records in which the official veterinarians have documented the most. Testicular size correlates with sperm production capacity and is an important factor for the prognostic assessment of breeding ability.^25^, ^26^ The significant effect of age at licensing on testicular size, with an average smallest size in the youngest and largest size in the oldest age group (Table S2), was to be expected, because testicular development is still ongoing at 2–3 years of age.^27^, ^28^ Testicular torsion was detected in 2.8% of the stallions. Testicles of young stallions are more predisposed to this condition than those of older stallions (≥4 years) due to their lack of stability.^25^ However, it usually remains in a subclinical stage, which is in line with the absence of notes of associated pain in our datasets.

Respiratory noise (5.1%) was less frequent than in studies in Thoroughbred or mixed populations (6.3%–10.9%).^24^, ^29^, ^30^, ^31^ Endoscopic examination of the respiratory tract is not an integral part of the standardised licensing examination for German Warmblood horses, becoming obligatory only if abnormal respiratory noise has been recognised. The most common result of the endoscopy was not surprisingly pharyngeal lymphoid hyperplasia (82.3%). This condition is described as reaching its highest prevalence of nearly 100% in 2‐year‐old horses.^32^, ^33^ The prevalence of recurrent laryngeal neuropathy cannot be determined from our dataset, as not all horses affected by laryngeal paralysis produce abnormal respiratory noise, and clinical signs are more likely to be observed during exercise.^14^, ^34^ Although the candidate stallions' examinations included exercise on the lunge and post‐exercise evaluation, false negative results cannot be excluded because the endoscopic examination was not performed in all stallions.

Cardiac abnormalities, mainly in the form of minor heart murmurs, were noted in 0.9% of the study population and this is in marked contrast to studies in other equine population which determined the prevalence of heart murmurs to be approximately 80% (*n* = 846 racehorses, mean age = 5.9 years; *n* = 822 Warmblood horses, mean age = 9.17 years).^35^, ^36^ The reason for this discrepancy remains speculative but may be due to differences in age and the level of training, as these factors may increase the occurrence of heart murmurs.^37^

Overjet is an exclusion criterion for licensing,^5^ as the condition has been described as to be determined by genetics to a relevant extent, requires enhanced life‐long dental care, and can lead to discomfort during feed intake in advanced stages.^38^, ^39^ However, overjet is tolerated in candidate stallions if it amounts to less than half a tooth width in the physiological head position. Overjet has been described as occurring in 2%–5% of all horses.^40^, ^41^, ^42^ Our population ranges below that (1.7% and only low‐grade degree), possibly due to high preselection.

Overall, the majority of findings in the clinical records of the young stallions appeared to be of minor clinical relevance. This is similar to the analysis of pre‐purchase examinations of National Hunt racehorses, in which trivial abnormal findings, such as skin lesions, greatly increased the proportion of horses with recorded abnormalities.^24^ More serious abnormalities were probably recognised earlier in the selection process, such that the affected stallions had already been excluded before the licensing examination.

Generalised linear models were applied to determine the role of environmental effects on the distributions of main clinical findings to account for the non‐normal and non‐linear nature of the target traits as well as robust interpretation of their distributions. Categorical traits with more than just two possible expressions are often regarded as quasi‐linear and analysed with linear models. We avoided such simplified modelling and consistently followed the threshold theory. Our hypothesis that the influence of the fixed effects tested was mostly minor was confirmed: No significant effects of age at licensing, season of birth, or year of licensing were found for most of the main clinical findings. The high level of homogeneity of the horse sample must be taken into account when interpreting these results, which prevents possible transfer to the whole Warmblood population. However, the analyses indicated a significant effect of the evaluator on several findings. This result is not surprising considering the probable differences in how the examination and documentation of findings were performed. Efforts should be made to further improve standardisation in the performance and documentation of the examination in order to refine the conditions for future comparative analyses.

A limitation of the present study is that the study population consisted of a homogeneous preselected group of horses and on average only 5% of stallions born in one given year are accepted to participate in the licensing process.^7^ Preselection is influenced by a variety of factors, including the breeders' ambitions and the breeding associations' assessment of the conformation, performance potential, and so forth. Accordingly, health‐related aspects play an important role, but are not the primary selection criterion of stallions for Warmblood breeding. Apart from the preselection, the sex might have influenced the results. Androgens can have a disease‐promoting effect, for example, on the musculoskeletal system.^43^ In general, the homogeneity of the population is not a limitation of the study itself. However, the German equine health database was created to provide sufficient data to represent the health status of the entire German Warmblood population which cannot be achieved with the data on which the current study is based.

Another main limitation of the study is that the retrospective data collection revealed different levels of detail in the descriptions of the findings. Including these descriptions in the database in a standardised way required interpretation by the first author and there is a possibility of misinterpretation in rare cases. A precise and standardised description of findings should be more widespread among evaluators to avoid this in the future. This would benefit not only data analysis but also the legal protection of veterinarians and communication with breeders, breeding associations, and future horse owners.

Although this study only focuses on the German horse‐breeding system, the study illustrates how national databases can be mined for useful descriptive data on the levels of health within specific populations. The study provides insight into the clinical status of candidate stallions for German Warmblood breeding in recent years. Although about half of the population had abnormalities documented, 51.9% of these stallions showed only one finding, and most of the findings in the young stallions seemed to be trivial. Thus, the future breeding stallion population appears, for the most part, to be clinically healthy. Nevertheless, the integration of health aspects in breeding should be strengthened. This study contributes to this, while continuous data collection and evaluation, improvement of the current database, and cooperation between all stakeholders are warranted for further progress.

## AUTHOR CONTRIBUTIONS

**Muriel Sarah Folgmann:** Conceptualization; writing – original draft; funding acquisition; investigation; methodology; data curation; project administration; visualization. **Kathrin Friederike Stock:** Conceptualization; writing – review and editing; methodology; software; data curation; validation. **Karsten Feige:** Supervision; project administration; conceptualization; writing – review and editing. **Uta Delling:** Supervision; writing – review and editing; conceptualization; funding acquisition; project administration.

## FUNDING INFORMATION

This study was funded by the foundation of H. Wilhelm Schaumann in the form of a doctoral scholarship.

## CONFLICT OF INTEREST STATEMENT

The authors declare no conflicts of interest.

## DATA INTEGRITY STATEMENT

Muriel Sarah Folgmann and Kathrin Friederike Stock had full access to all the data in the study and take responsibility for the integrity of the data and the accuracy of data analysis.

## ETHICAL ANIMAL RESEARCH

Research ethics committee oversight is not required by this journal: The study was based on retrospective data of licensing examinations.

## INFORMED CONSENT

The licensing examination data are the property of the respective horse‐breeding associations and written consent for this study was obtained. Owners of stallions presented for licensing sign an agreement with the respective breeding association, which included permission for the scientific use of the collected health data by third parties. Official veterinarians associations sign a data protection declaration as part of the registration process for using the German equine health database.

## PEER REVIEW

The peer review history for this article is available at https://www.webofscience.com/api/gateway/wos/peer-review/10.1111/evj.14474.

## Supporting information


**Figure S1.** Protocol for clinical examinations prior to stallion licensing according to the German Breeding Association Regulation. The protocol has been translated from the original German by the authors with the authorisation of the FN. The term ‘overjet’ describes the projection of the maxillary incisors labial to their antagonists in a horizontal direction. NAF, no abnormal findings; Abn., abnormality; UELN, universal equine life number; EVA, equine viral arteritis.

**Table S1.** Results of generalised linear models with Chi‐squares (χ^2^) and error probabilities (P) for the fixed effects modelled and main clinical findings from 1655 examinations of candidate stallions prior to licensing for German Warmblood breeding in 2018–2020. Table legend: Mc/Mt., metacarpal/metatarsal bone. Levels of significance: ***p < 0.001; **p < 0.01; *p < 0.05; ^+^ *p* < 0.10 (tendency).

**Table S2.** Results of generalised linear models with number of observations (n), least square mean estimates (LSM) transformed from underlying to observed scale, their standard errors (SE) and 95%‐confidence intervals (95% CI with lower limit (LL) and upper limit (UL)) for fixed effects with significant influence on the distribution of main clinical findings from 1655 examinations of candidate stallions prior to licensing for German Warmblood breeding in 2018–2020. Table legend: Mc/Mt., metacarpal/metatarsal bone. n.e., not estimable. ^1^The figures in the individual rows refer to the following testicular sizes: first row = goose egg sized, second row = duck to goose egg sized, third row = duck egg sized, fourth row = chicken to duck egg sized. Intermediate sizes occur when a horse had testicles of two different sizes. Data from original evaluators 6, 7, and 8 were grouped together into evaluator 99 because of the low number of records by these examiners (<7% of total).

## Data Availability

The data that support the findings of this study are available from the corresponding author upon reasonable request: Open sharing exemption granted by editor for this descriptive retrospective clinical report.
