# Supplementary material for: Clinical findings of candidate stallions presented for licensing at all German Warmblood horse‐breeding associations in 2018–2020
Source: Equine Vet J. 2025 Jan 22;57(6):1584–91. doi: 10.1111/evj.14474 (PMC12508275; doi:10.1111/evj.14474)
Supplement: Supplementary file 1 — Figure S1. Protocol for clinical examinations prior to stallion licensing according to the German Breeding Association Regulation. The protocol has been translated from the original German by the authors with the authorisation of the FN. The term ‘overjet’ describes the projection of the maxillary incisors labial to their antagonists in a horizontal direction. NAF, no abnormal findings; Abn., abnormality; UELN, universal equine life number; EVA, equine viral arteritis. [file EVJ-57-1584-s003.pdf]

Figure S1:

Protocol of the Clinical Examination of a Stallion

01	Owner			
02	Horse name		Born:	
03	UELN		Chip number:	
			☐ Signalement checked	
04	Colour		☐ Sire	☐ Dam sire
05	Previous diseases/surgeries		☐ No<br>☐ Yes	☐ Owner declaration available
	Medications within the last 6 weeks:			
06	Vaccination according to horse passport		☐ Influenza<br>☐ Herpes	☐ Tetanus<br>☐ Other
07	Witness to the examination:			

Examination

08	Care and nutritional status		☐ NAF	☐ Abn.:
09	Skin and scars (e.g. surgery scars, umbilical hernia, etc.)		☐ NAF	☐ Abn.:
10	Adspection and palpation of head, neck, trunk area, back		☐ NAF	☐ Abn.:
11	Incisors		☐ NAF	☐ Abn.:
	Overjet (less than 50% in friction and physiological head position)		☐ No	☐ Yes
12	Eyes (in darkened room)		☐ NAF	☐ Abn.:
13	Testicles			
	Consistency	right	☐ elastic	☐ soft
		left	☐ elastic	☐ soft
	Size	right	☐ goose egg<br>☐ chicken egg	☐ duck egg<br>☐ < chicken egg
		left	☐ goose egg<br>☐ chicken egg	☐ duck egg<br>☐ < chicken egg
	Specifics:			
14	Prepuce, Scrotum		☐ NAF	☐ Abn.:

Equine Veterinary Journal BEVA

15	Circulatory and respiratory system at rest (incl. auscultation)	☐ NAF	☐ Abn.:
16	Spontaneous coughing	☐ not present	☐ present
17	Adspection and palpation of limbs	LF<br>LH	RF<br>RH
18	Hoof conformation, hoof, hoof shape	☐ NAF	☐ Abn.:
19	Shoeing<br>Specifics:	☐ none	☐ front<br>☐ hind
20	Evaluation at walk and trot in hand on straight line on firm ground	☐ NAF	☐ Abn.:
20a	Trotting on the circle on soft and firm ground on both hands	☐ NAF	☐ Abn.:
20b	Backing up	☐ NAF	☐ Abn.:
20c	Tight turns	☐ NAF	☐ Abn.:
21	Neurological disorders (signs of ataxia, stringhalt or shivering)	☐ No	☐ Yes
22	Examination during excercise until onset of intensive breathing	☐ NAF	☐ Abn.:
23	Respiratory noise during and after excercise	☐ none<br>☐ inspiratory	☐ exspiratory
23a	Laryngoscopy under sedation (obligatory if respiratory noise is present)<br>Larynx (movement of arytenoid cartilages)<br>Other findings:	☐ synchronous with complete abduction of arytenoid cartilages<br>☐ asynchronous with complete abduction of arytenoid cartilages<br>☐ asynchronous with incomplete abduction of arytenoid cartilages	
24	Auscultation of heart and lungs after excercise	☐ NAF	☐ Abn.:
25	Blood sample for EVA	☐ Date:	
26	Further examinations:		
	Follow-up examinations required	☐ No	☐ Yes, because of

Date of Examination	Name of Veterinarian	Signature of Veterinarian

Figure S1: Protocol for clinical examinations prior to stallion licensing according to the German Breeding Association Regulation.

The protocol has been translated from the original German by the authors with the authorisation of the FN.

The term ‘overjet’ describes the projection of the maxillary incisors labial to their antagonists in a horizontal direction.

NAF, no abnormal findings; Abn., abnormality; UELN, universal equine life number; EVA, equine viral arteritis
