# Supplementary material for: Clinical findings of candidate stallions presented for licensing at all German Warmblood horse‐breeding associations in 2018–2020
Source: Equine Vet J. 2025 Jan 22;57(6):1584–91. doi: 10.1111/evj.14474 (PMC12508275; doi:10.1111/evj.14474)
Supplement: Supplementary file 2 — Table S1. Results of generalised linear models with Chi‐squares (χ2) and error probabilities (P) for the fixed effects modelled and main clinical findings from 1655 examinations of candidate stallions prior to licensing for German Warmblood breeding in 2018–2020. Table legend: Mc/Mt., metacarpal/metatarsal bone. Levels of significance: ***p < 0.001; **p < 0.01; *p < 0.05; + p < 0.10 (tendency). [file EVJ-57-1584-s002.pdf]

Equine Veterinary Journal BEVA

Table S1: Results of generalised linear models with Chi-squares (χ^2) and error probabilities (P) for the fixed effects modelled and main clinical findings from 1655 examinations of candidate stallions prior to licensing for German Warmblood breeding in 2018–2020.

Fixed effect / Clinical finding	Season of birth		Age at licensing		Year of licensing		Evaluator	
	χ2	P	χ2	P	χ2	P	χ2	P
exostosis/swelling/filling (limbs)	7.65	0.105	2.40	0.301	0.31	0.867	17.26	0.004**
- front limbs	9.61	0.048*	4.68	0.096+	1.38	0.503	15.67	0.008**
- hind limbs	0.34	0.987	0.10	0.953	0.70	0.704	2.00	0.849
- Mc/Mt/splint bone	6.89	0.142	4.68	0.096+	6.44	0.040*	32.13	< 0.001***
- Mc/Mt/splint bone front	6.09	0.193	4.84	0.089+	5.95	0.051+	23.24	< 0.001***
injuries/scars	11.33	0.023*	1.94	0.379	2.65	0.265	15.12	0.009**
- distal limb	12.26	0.016*	4.45	0.108	2.82	0.244	14.70	0.012*
- front limbs	1.74	0.784	1.66	0.435	4.86	0.088+	9.89	0.078+
- hind limbs	14.71	0.005**	1.28	0.526	2.27	0.321	10.68	0.058+
- fetlock/pastern	7.03	0.134	3.14	0.208	0.73	0.694	11.52	0.042*
abnormal limb conformation	5.81	0.214	6.06	0.048*	0.34	0.843	35.03	< 0.001***
- front limbs	4.65	0.326	1.46	0.481	0.78	0.677	32.71	< 0.001***
- toe	5.7	0.223	2.45	0.293	0.27	0.873	34.20	< 0.001***
- toe front	4.31	0.365	0.30	0.859	1.13	0.567	30.91	< 0.001***
respiratory noise	3.91	0.42	6.96	0.031*	0.41	0.813	70.54	< 0.001***
- inspiratory respiratory noise	3.43	0.488	7.14	0.028*	0.23	0.890	71.89	< 0.001***
testicular size	3.90	0.419	9.49	0.009**	1.55	0.461	27.91	< 0.001***

Mc/Mt, metacarpal / metatarsal bone

levels of significance: *** = $P < 0.001$, ** = $P < 0.01$, * = $P < 0.05$, + = $P < 0.10$ (tendency)
