# Supplementary material for: Clinical findings of candidate stallions presented for licensing at all German Warmblood horse‐breeding associations in 2018–2020
Source: Equine Vet J. 2025 Jan 22;57(6):1584–91. doi: 10.1111/evj.14474 (PMC12508275; doi:10.1111/evj.14474)
Supplement: Supplementary file 3 — Table S2. Results of generalised linear models with number of observations (n), least square mean estimates (LSM) transformed from underlying to observed scale, their standard errors (SE) and 95%‐confidence intervals (95% CI with lower limit (LL) and upper limit (UL)) for fixed effects with significant influence on the distribution of main clinical findings from 1655 examinations of candidate stallions prior to licensing for German Warmblood breeding in 2018–2020. Table legend: Mc/Mt., metacarpal/metatarsal bone. n.e., not estimable. 1The figures in the individual rows refer to the following testicular sizes: first row = goose egg sized, second row = duck to goose egg sized, third row = duck egg sized, fourth row = chicken to duck egg sized. Intermediate sizes occur when a horse had testicles of two different sizes. Data from original evaluators 6, 7, and 8 were grouped together into evaluator 99 because of the low number of records by these examiners (<7% of total). [file EVJ-57-1584-s001.pdf]

Table S2: Results of generalised linear models with number of observations (n), least square mean estimates (LSM) transformed from underlying to observed scale, their standard errors (SE) and 95%-confidence intervals (95% CI with lower limit (LL) and upper limit (UL)) for fixed effects with significant influence on the distribution of main clinical findings from 1655 examinations of candidate stallions prior to licensing for German Warmblood breeding in 2018–2020.

Finding	Effect level	n	LSM	SE	95 %-CI	
Fixed effect					LL	UL
**Exostosis/swelling/filling (limbs)**						
evaluator	1	314	0.14	0.54	0.100	0.196
	2	230	0.21	0.55	0.150	0.289
	3	221	0.14	0.55	0.089	0.197
	4	293	0.15	0.55	0.100	0.203
	5	317	0.11	0.54	0.076	0.157
	99	280	0.22	0.54	0.171	0.284
- front limbs						
season of birth	Nov–Feb	121	0.19	0.57	0.114	0.288
	March	315	0.13	0.55	0.083	0.185
	April	514	0.12	0.54	0.089	0.164
	May	528	0.07	0.54	0.049	0.106
	Jun–Oct	177	0.08	0.56	0.041	0.134
evaluator	1	314	0.10	0.55	0.061	0.143
	2	230	0.16	0.55	0.105	0.234
	3	221	0.10	0.56	0.057	0.152
	4	293	0.10	0.55	0.063	0.152
	5	317	0.08	0.55	0.047	0.115
	99	280	0.17	0.54	0.123	0.227
- Mc/Mt/splint bone						
year of licensing	2018	561	0.08	0.54	0.058	0.119
	2019	599	0.13	0.54	0.094	0.166
	2020	495	0.13	0.54	0.094	0.175
evaluator	1	314	0.07	0.55	0.043	0.113
	2	230	0.18	0.55	0.117	0.249
	3	221	0.08	0.56	0.045	0.132
	4	293	0.12	0.55	0.076	0.172
	5	317	0.07	0.55	0.046	0.113
	99	280	0.20	0.54	0.148	0.257

- Mc/Mt/splint bone front						
evaluator	1	314	0.05	0.56	0.027	0.088
	2	230	0.13	0.56	0.083	0.203
	3	221	0.07	0.56	0.039	0.123
	4	293	0.09	0.55	0.053	0.137
	5	317	0.07	0.55	0.040	0.104
	99	280	0.16	0.54	0.116	0.218
**Injuries/scars**						
season of birth	Nov–Feb	121	0.10	0.57	0.052	0.182
	March	315	0.17	0.55	0.115	0.231
	April	514	0.15	0.54	0.108	0.192
	May	528	0.11	0.54	0.081	0.154
	Jun–Oct	177	0.06	0.56	0.031	0.110
evaluator	1	314	0.13	0.54	0.090	0.183
	2	230	0.12	0.55	0.072	0.177
	3	221	0.14	0.55	0.090	0.197
	4	293	0.11	0.55	0.072	0.163
	5	317	0.06	0.55	0.036	0.095
	99	280	0.14	0.54	0.101	0.196
- distal limb						
season of birth	Nov–Feb	121	0.11	0.57	0.057	0.194
	March	315	0.16	0.55	0.109	0.225
	April	514	0.14	0.54	0.104	0.186
	May	528	0.09	0.54	0.061	0.125
	Jun–Oct	177	0.06	0.57	0.027	0.102
evaluator	1	314	0.13	0.55	0.087	0.181
	2	230	0.11	0.56	0.064	0.166
	3	221	0.13	0.55	0.086	0.193
	4	293	0.09	0.55	0.056	0.138
	5	317	0.06	0.55	0.036	0.095
	99	280	0.14	0.54	0.096	0.190
- hind limb						
season of birth	Nov–Feb	121	0.05	0.59	0.018	0.112
	March	315	0.11	0.55	0.067	0.164
	April	514	0.11	0.54	0.078	0.152
	May	528	0.08	0.54	0.053	0.116
	Jun–Oct	177	0.03	0.58	0.010	0.068
**Abnormal limb conformation**						

age at licensing	< 30M	677	0.06	0.55	0.034	0.089
	30–36M	897	0.04	0.54	0.028	0.062
	> 36M	81	0.13	0.58	0.060	0.229
evaluator	1	314	0.03	0.57	0.013	0.063
	2	230	0.15	0.56	0.088	0.224
	3	221	0.05	0.57	0.021	0.093
	4	293	0.14	0.55	0.090	0.208
	5	317	0.05	0.56	0.027	0.086
	99	280	0.06	0.56	0.032	0.098
- front limb						
evaluator	1	314	0.03	0.57	0.010	0.055
	2	230	0.11	0.57	0.062	0.187
	3	221	0.04	0.57	0.017	0.081
	4	293	0.12	0.56	0.069	0.179
	5	317	0.04	0.56	0.020	0.073
	99	280	0.03	0.57	0.013	0.061
- toe						
evaluator	1	314	0.02	0.58	0.009	0.053
	2	230	0.13	0.56	0.073	0.201
	3	221	0.04	0.57	0.018	0.085
	4	293	0.13	0.56	0.078	0.189
	5	317	0.04	0.56	0.022	0.076
	99	280	0.06	0.56	0.030	0.095
- toe front						
evaluator	1	314	0.02	0.58	0.006	0.042
	2	230	0.09	0.57	0.044	0.157
	3	221	0.03	0.58	0.012	0.070
	4	293	0.09	0.56	0.051	0.155
	5	317	0.03	0.57	0.014	0.061
	99	280	0.03	0.57	0.011	0.056
**Respiratory noise**						
age at licensing	< 30M	677	0.02	0.56	0.011	0.045
	30–36M	897	0.04	0.54	0.026	0.061
	> 36M	81	n.e.			
- inspiratory respiratory noise						
age at licensing	< 30M	677	0.02	0.56	0.010	0.042
	30–36M	897	0.04	0.54	0.025	0.059
	> 36M	81	n.e.			

**Testicular size** [1]						
age at licensing	< 30M	677	0.55	0.53	0.500	0.607
			0.57	0.53	0.518	0.623
			0.97	0.54	0.957	0.981
			0.98	0.54	0.971	0.989
	30–36M	897	0.63	0.52	0.593	0.670
			0.65	0.52	0.608	0.683
			0.98	0.53	0.974	0.988
			0.99	0.54	0.983	0.993
	> 36M	81	0.72	0.56	0.614	0.812
			0.73	0.56	0.625	0.820
			0.99	0.57	0.979	0.996
			0.99	0.57	0.986	0.998
evaluator	1	314	0.61	0.54	0.548	0.678
			0.63	0.54	0.564	0.691
			0.98	0.54	0.968	0.988
			0.99	0.55	0.978	0.993
	2	230	0.68	0.54	0.601	0.747
			0.69	0.54	0.615	0.759
			0.99	0.55	0.977	0.993
			0.99	0.55	0.985	0.996
	3	221	0.54	0.54	0.466	0.613
			0.55	0.54	0.478	0.624
			0.97	0.54	0.950	0.981
			0.98	0.55	0.965	0.989
	4	293	0.61	0.54	0.537	0.674
			0.62	0.54	0.548	0.683
			0.98	0.54	0.966	0.988
			0.99	0.55	0.976	0.993
	5	317	0.74	0.53	0.678	0.788
			0.75	0.54	0.692	0.799
			0.99	0.54	0.986	0.995
			1.00	0.55	0.991	0.998
	99	280	0.64	0.53	0.579	0.704
			0.66	0.54	0.601	0.722
			0.98	0.54	0.973	0.990
			0.99	0.55	0.983	0.995

Mc/Mt, metacarpal / metatarsal bone

n.e., not estimable

[1]The figures in the individual rows refer to the following testicular sizes: first row = goose egg sized, second row = duck to goose egg sized, third row = duck egg sized, fourth row = chicken to duck egg sized. Intermediate sizes occur when a horse had testicles of two different sizes.

Data from original evaluators 6, 7, and 8 were grouped together into evaluator 99 due to fewer records (< 7%).
